# Pelvic lymph node dissection and its extent on survival benefit in prostate cancer patients with a risk of lymph node invasion >5%: a propensity score matching analysis from SEER database

**DOI:** 10.1038/s41598-019-54261-4

**Published:** 2019-11-29

**Authors:** Junru Chen, Zhipeng Wang, Jinge Zhao, Sha Zhu, Guangxi Sun, Jiandong Liu, Haoran Zhang, Xingming Zhang, Pengfei Shen, Ming Shi, Hao Zeng

**Affiliations:** Department of Urology, Institute of Urology, West China Hospital, Sichuan University, Chengdu, 610041 China

**Keywords:** Urological cancer, Prostate

## Abstract

Pelvic lymph node dissection (PLND) represents the gold standard for nodal staging in PCa and is recommended for patients with a probability of lymph node invasion (LNI) >5%. However, the therapeutic role of PLND and its extent remains a debate. In this study, data of 20,668 patients treated with radical prostatectomy (RP) with and without PLND from SEER database between 2010 and 2015 were retrospectively analyzed. All patients had a risk of LNI >5% according to 2012-Briganti nomogram. Propensity score matching (PSM) was performed to balance baseline characteristics between patients with and without PLND. Kaplan-Meier curves and Cox regression were used to evaluate the impacts of the PLND and its extent on cancer-specific survival (CSS) and overall survival (OS). In overall cohort, patients with PLND were associated with more aggressive clinicopathologic characteristics and had poorer survival compared to those without PLND (5-year CSS rate: 98.4% vs. 99.7%, p < 0.001; 5-year OS rate: 96.3% vs. 97.8%, p < 0.001). In the post-PSM cohort, no significant difference in survival was found between patients with and without PLND (5-year CSS rate: 99.4% vs. 99.7%, p = 0.479; 5-year OS rate: 97.3% vs. 97.8%, p = 0.204). In addition, the extent of PLND had no impact on prognosis (all p > 0.05). Subgroup analyses reported similar negative findings. In conclusion, neither PLND nor its extent was associated with survival in North American patients with a risk of LNI >5%. The cut-off point of 5% probability of LNI might be too low to show benefits in survival in patients underwent PLND.

## Introduction

With the widespread use of prostate specific antigen (PSA) screening, prostate cancer (PCa) has become the most common solid malignancy in men in North America^1^. According to latest guidelines, radical prostatectomy (RP) is recommended as one of the curable therapies for patients with localized PCa^2^. Pelvic lymph node dissection (PLND) is an important component in comprehensive RP and represents the gold standard for nodal staging in PCa^3^. Despite the essential role of PLND in PCa staging, its therapeutic value is still obscure^3^. Furthermore, it is also associated with higher risk of perioperative complications such as increased blood loss, lymphoceles and thromboembolic events^4,5^. Therefore, several tools have been established to predict the risk of lymph node invasion (LNI) to select potential optimal candidates who could benefit from PLND^6^. According to European Association of Urology (EAU) guidelines, PLND is recommended for patients with a probability of LNI over 5% based on 2012-Briganti nomogram^2,7^. Contemporarily, in real world clinical practice, about 30% of patients with a relatively high risk of LNI according to different predicting tools would not receive PLND at RP in the US^8^. Thus, with the improved PCa imaging and multimodal treatment approaches, it is of great necessity to comprehensively evaluate the survival benefit of PLND in patients with localized PCa.

In the present study, we aimed to investigate the therapeutic role of PLND and its extent in North American patients with a risk of LNI >5%.

## Results

### Patients characteristics

In total, 271,662 patients with PCa as the only one primary tumor were retrieved from SEER database between 2010 and 2015, 193,693 patients were excluded based on inclusion and exclusion criteria, 30,933 patients were excluded for missing data. Finally, 20,668 patients underwent RP whose probabilities of LNI were >5% in SEER database were included with a median age of 63 years. Median follow-up period for entire cohort was 32 months. Baseline patient characteristics for overall cohort and post-PSM cohort were shown in Table 1. For the whole cohort, 16,401 (79.4%) patients were treated with RP plus PLND and 4,267 (20.6%) patients received RP alone as primary treatment. The median NRN for patients underwent PLND was 6 and the 75th percentile was 11. Among patients with PLND, 11,648 (71.0%) received a more extensive PLND (NRN ≥11), 1,681 (10.2%) had LNI. Patients who underwent PLND were associated with higher cT/pT stage, increased PSA values, higher GS at biopsy/RP and more high-risk PCa (D’ Amico stratification) compared to those without PLND. PSM resulted in 4,267 patients in each group. After PSM, the baseline characteristics were well balanced in patients with and without PLND.

**Table 1:** Baseline characteristics of patients with and without PLND in overall cohort and propensity-score matched cohort.

Characteristics	Overall cohort			Propensity-score matched cohort		
	No PLND (n = 4267)	PLND (n = 16401)	p value	No PLND (n = 4267)	PLND (n = 4267)	p value
**Age**, **years**, **n (%)**						
<70	3687 (86.4%)	14084 (85.9%)	0.37	3687 (86.4%)	3716 (87.1%)	0.355
≥70	580 (13.6%)	2317 (14.1%)		580 (13.6%)	551 (12.9%)	
**Race**, **n (%)**						
Black	647 (15.2%)	2290 (14.0%)	<0.001	647 (15.2%)	659 (15.4%)	0.809
White	3418 (80.1%)	12852 (78.4%)		3418 (80.1%)	3396 (79.6%)	
Others	202 (4.7%)	1259 (7.6%)		202 (4.7%)	212 (5.0%)	
**D’Amico risk stratification**, **n (%)**						
Low	366 (8.6%)	369 (2.3%)	<0.001	366 (8.6%)	364 (8.5%)	0.975
Intermediate	2658 (62.3%)	7463 (45.5%)		2658 (62.3%)	2668 (62.5%)	
High	1243 (29.1%)	8569 (52.2%)		1243 (29.1%)	1235 (29.0%)	
**Clinical T stage**, **n (%)**						
≤T2	4080 (95.6%)	15289 (93.2%)	<0.001	4080 (95.6%)	4051 (94.9%)	0.139
≥T3	187 (4.4%)	1112 (6.8%)		187 (4.4%)	216 (5.1%)	
**PSA**, **ng/ml**, **n (%)**						
≤20	4039 (94.7%)	14462 (88.2%)	<0.001	4039 (94.7%)	4020 (94.2%)	0.370
>20	228 (5.3%)	1939 (11.8%)		228 (5.3%)	247 (5.8%)	
**Pathological T stage**, **n (%)**						
≤T2	2837 (66.5%)	8261 (50.4%)	<0.001	2837 (66.5%)	2811 (65.9%)	0.552
≥T3	1430 (33.5%)	8140 (49.6%)		1430 (33.5%)	1456 (34.1%)	
**Gleason score at biopsy**, **n (%)**						
≤7	3649 (85.5%)	10268 (62.6%)	<0.001	3649 (85.5%)	3668 (86.0%)	0.556
≥8	618 (14.5%)	6133 (37.4%)		618 (14.5%)	599 (14.0%)	
**Gleason score at RP**, **n (%)**						
≤7	3766 (88.3%)	12093 (73.7%)	<0.001	3766 (88.3%)	3755 (88.0%)	0.713
≥8	501 (11.7%)	4308 (26.3%)		501 (11.7%)	512 (12.0%)	
**Number of removed nodes**, **median (range)**						
		6 (1–70)			6 (1–66)	
**Lymph node invasion, n (%)**						
		1681 (10.2%)			250 (5.9%)	

### Survival analyses

**Figure 1:**
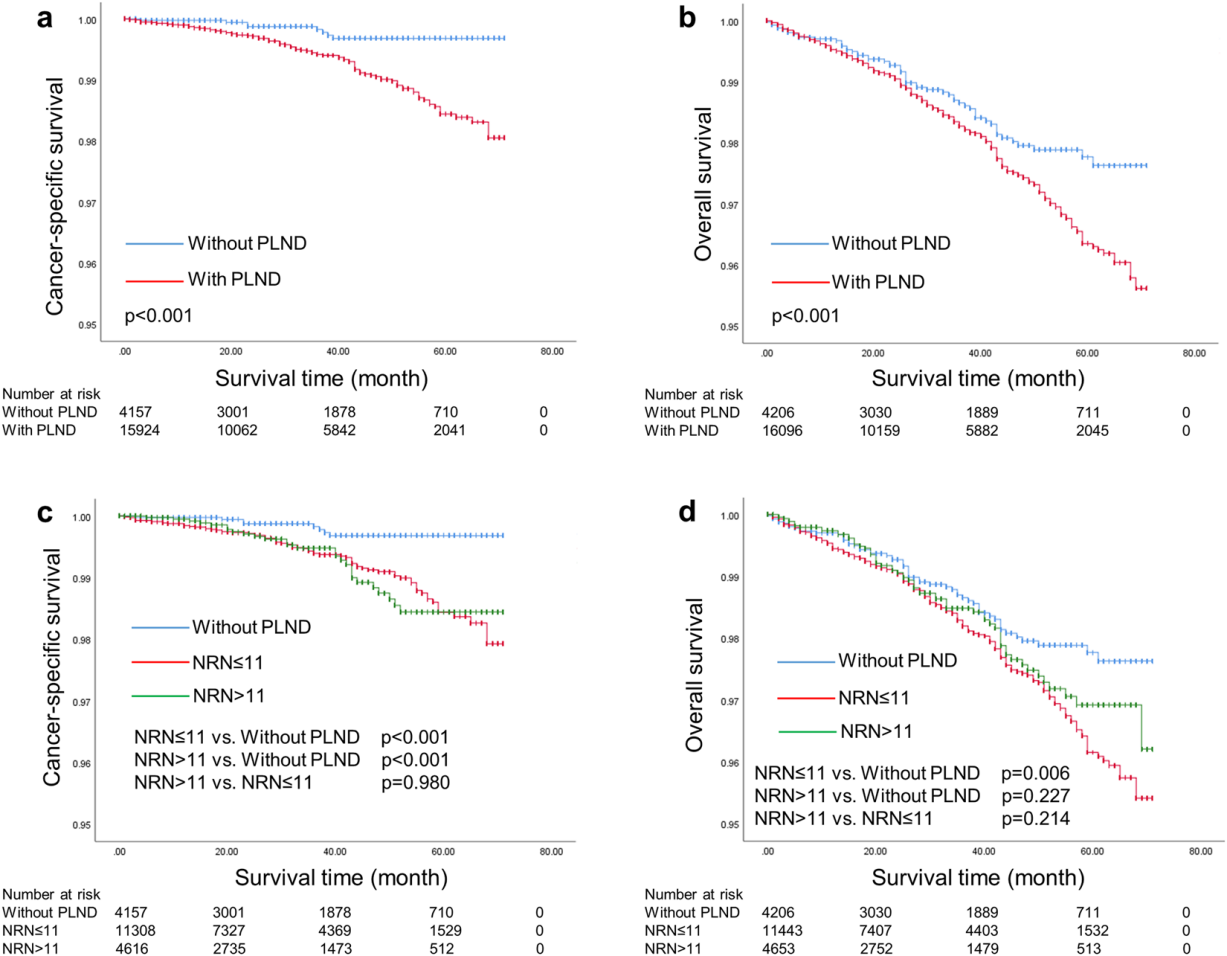
(**a**,**b**) Kaplan-Meier curves of cancer-specific survival and overall survival for patients with and without PLND in the entire cohort; (**c**,**d**) Kaplan-Meier curves of cancer-specific survival and overall survival for patients with different extents of PLND in the entire cohort.

For the entire cohort, as shown in Fig. 1a,b, patients with PLND was associated with poorer survival than those without PLND (5-year CSS rate: 98.4% vs. 99.7%, p < 0.001; 5-year OS rate: 96.3% vs. 97.8%, p < 0.001). After stratifying patients according to extent of PLND (Fig. 1c,d), patients without PLND had better survival outcomes compared to those with less extensive and more extensive PLND (5-year CSS rate: 99.7% vs. 98.4% vs. 98.4%, respectively, p < 0.001; 5-year OS rate: 97.8% vs. 96.2% vs. 96.9%, respectively, p = 0.017).

**Figure 2:**
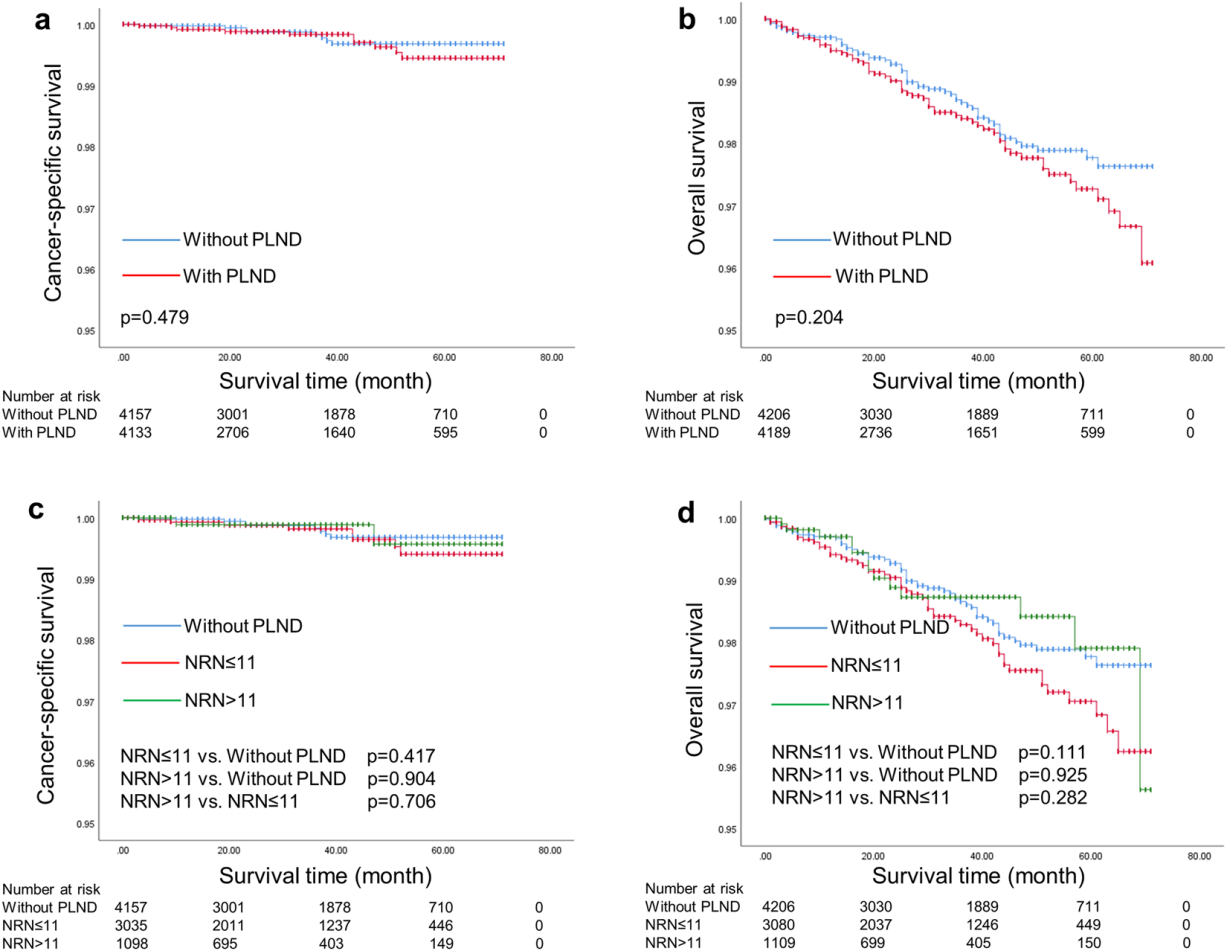
(**a**,**b**) Kaplan-Meier curves of cancer-specific survival and overall survival for patients with and without PLND in the post-PSM cohort; (**c**,**d**) Kaplan-Meier curves of cancer-specific survival and overall survival for patients with different extents of PLND in the post-PSM cohort.

**Figure 3:**
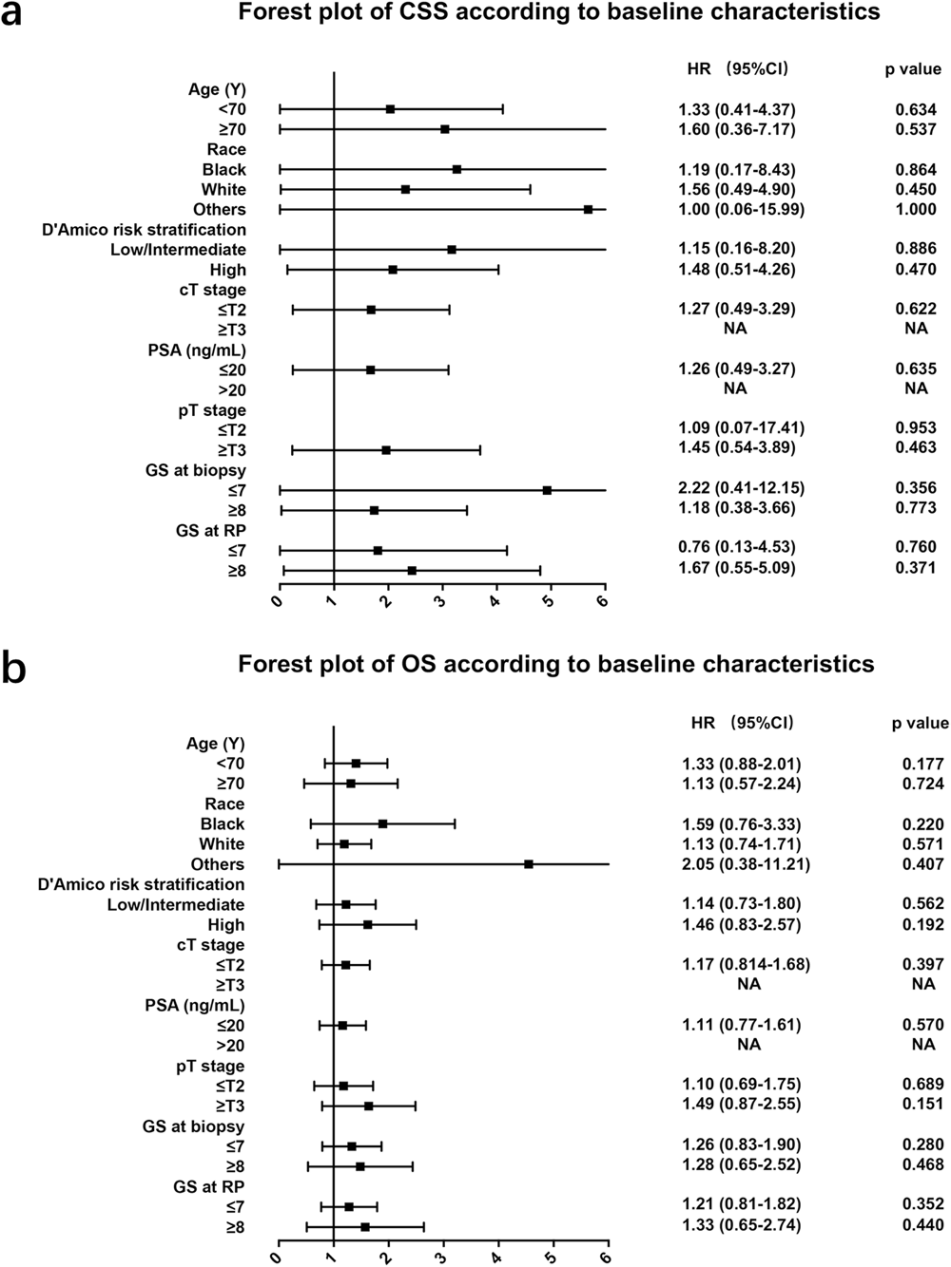
(**a**) Forest plot showing the prognostic significance of PLND in predicting cancer-specific survival for patients with different baseline characteristics in the post-PSM cohort. (**b**) Forest plot showing the prognostic significance of PLND in predicting overall survival for patients with different baseline characteristics in the post-PSM cohort.

In the post-PSM cohort, as shown in Fig. 2a,b, no significant difference of survival outcomes was found in patients with and without PLND (5-year CSS rate: 99.4% vs. 99.7%, p = 0.479; 5-year OS rate: 97.3% vs. 97.8%, p = 0.204). Further analyses according to extent of PLND were also performed (Fig. 2c,d). Kaplan-Meier plots demonstrated 5-year CSS rates of 99.7%, 99.4% and 99.6% for patients without PLND, with less extensive PLND and with more extensive PLND, respectively (p = 0.714). In addition, Kaplan-Meier plots showed similar 5-year OS rates for patients without PLND, with less extensive PLND and with more extensive PLND (97.8% vs. 97.0% vs. 97.9%, respectively, p = 0.234). Subgroup analyses based on baseline characteristics showed that PLND provided no significant survival benefit in patients with different baseline clinicopathologic features (Fig. 3). Patients underwent more extensive PLND had similar prognosis compared to those without PLND in subgroup analyses (data not shown).

As shown in Table 2, Cox regression analyses indicated that PLND (no matter the extent of PLND) was not associated with CSS and OS in patients with localized PCa. Multivariate analyses demonstrated that age ≥70, high-risk PCa, pT stage ≥3 and GS ≥8 were independent predictors for poor prognosis in patients with localized PCa.

**Table 2:** Univariate and Multivariate analyses of cancer-specific survival and overall survival for patients in post-propensity score matching cohort.

	CSS				OS			
	Univariate analyses		Multivariate analyses		Univariate analyses		Multivariate analyses	
	HR (96% CI)	p value	HR (96% CI)	p value	HR (96% CI)	p value	HR (96% CI)	p value
**Age (years)**								
≥70 vs. <70	4.57 (1.77–11.78)	0.002	4.65 (1.31–16.53)	0.005^*^	2.57 (1.73–3.83)	<0.001	2.64 (1.77–3.95)	<0.001^##^
**Race**								
White vs. Black	0.55 (0.18–1.72)	0.306			0.57 (0.38–0.87)	0.009	0.52 (0.34–0.79)	0.002^##^
Other vs. Black	1.51 (0.28–8.25)	0.633	*		0.64 (0.27–1.54)	0.320	0.55 (0.23–1.34)	0.189^##^
**Lymph node dissection**								
Yes vs. No	1.40 (0.55–3.54)	0.481			1.26 (0.88–1.79)	0.205		
NRN ≤ 10 vs. NRN = 0	1.50 (0.56–3.98)	0.421			1.35 (0.93–1.96)	0.112		
NRN > 10 vs. NRN = 0	1.11 (0.24–5.21)	0.899			0.97 (0.53–1.77)	0.927		
NRN continuously coded	1.03 (0.97–1.09)	0.309			1.01 (0.98–1.04)	0.572		
**Lymph node invasion**								
Yes vs. No	5.04 (1.07–23.79)	0.041	3.16 (0.66–15.13)	0.150^*^	1.25 (0.45–3.42)	0.671		
**D’Amico risk stratification**								
High vs. Low/Intermediate	9.00 (2.96–27.35)	<0.001	8.76 (1.83–41.84)	0.007^*^	1.67 (1.17–2.40)	0.005	1.61 (1.12–2.31)	0.010^##^
**PSA**, **ng/ml**								
>20 vs. ≤20	1.22 (0.16–9.13)	0.850			1.77 (0.93–3.37)	0.085		
**Clinical T stage**								
≥T3 vs. ≤T2	1.22 (0.16–9.16)	0.848			1.25 (0.58–2.68)	0.565		
**Gleason score at biopsy**								
≥8 vs. ≤7	14.03 (5.26–37.43)	<0.001	4.00 (2.49–6.41)	<0.001^**^	2.57 (1.73–3.82)	<0.001	2.32 (1.56–3.46)	<0.001^^^
**Pathological T stage**								
≥T3 vs. ≤T2	17.07 (3.93–74.26)	<0.001	8.27 (1.83–37.38)	0.006^#^	1.62 (1.14–2.32)	0.007	1.30 (0.90–1.89)	0.162^^^^
**Gleason score at RP**								
≥8 vs. ≤7	21.39 (7.62–60.05)	<0.001	11.01 (3.80–31.87)	<0.001^#^	2.57 (1.70–3.87)	<0.001	2.13 (1.39–3.28)	0.001^^^^

*Adjusted for: age, lymph node invasion and D’Amico risk stratification.

**Adjusted for: age, lymph node invasion and Gleason score at biopsy.

^#^Adjusted for: age, lymph node invasion, pathological T stage and Gleason score at RP.

^##^Adjusted for: age, race and D’Amico risk stratification.

^^^Adjusted for: age, race and Gleason score at biopsy.

^^^^Adjusted for: age, race, pathological T stage and Gleason score at RP.

## Discussion

LNI is a poor prognosticator for survival in patients with localized PCa^9,10^. Currently, PLND represents the most accurate procedure to detect the presence of LNI in men with PCa and identify candidates for adjuvant therapies. In theory, PLND could reduce recurrence as well as provide potential survival benefits by removing micro-metastases in lymph nodes. However, no conclusive evidence has verified the survival benefit of PLND in localized PCa^3^. Due to potential complications and unclear therapeutic value, PLND omission is also important for selected patients. A recent study compared four widespread preoperative nomograms and recommended the Cagiannos and the 2012-Briganti nomograms as the best tools for prediction of LNI before RP in North American population^6^. By far, whether patients suggested by these nomograms to receive PLND could gain survival benefit from this procedure remains unknown.

In the present study, we firstly investigated the therapeutic role of PLND and its extent in North American patients with a risk of LNI >5% based on 2012-Briganti nomogram. In the whole cohort, patients with PLND harbored more aggressive clinicopathologic features and had statistically poorer CSS and OS compared to those without PLND. This result was similar to Boehm’s study, which could be explained by obvious baseline differences^11^. After PSM, baseline characteristics were well balanced and no significant difference in survival was found between men with and without PLND. In addition, this negative result was also observed in various subgroups. Several previous studies investigating the therapeutic utility of PLND in patients with localized PCa have yielded similar results^12,13^. Chang and colleagues conducted a nested, case-control, matched study and demonstrated no statistical difference in CSS in patients who underwent PLND compared to those who did not^12^. Porter *et al*. reviewed data of 752 patients in the USA and reported that PLND status was not associated with CSS^13^. Interestingly, Pokala and colleagues analyzed long-term outcomes in patients with high-grade PCa and found that patients with PLND had shorter CSS than patients without PLND^14^. However, no obvious difference in OS was observed between two groups.

Recently, several studies have indicated that extended PLND (ePLND) could improve the detection of LNI in PCa compared to limited PLND (lPLND) or standard PLND (sPLND)^15–17^. Thus, current guidelines recommend to perform ePLND when PLND is indicated. However, controversy exists as to the therapeutic benefit of ePLND in clinical practice. According to our results, we failed to find any evidence of the beneficial influence of PLND extent on survival in patients with localized PCa. A recent systematic review and meta-analysis involving seven studies demonstrated that ePLND could provide oncological benefit for biochemical recurrence-free survival compared to sPLND^18^. Nevertheless, Nyushko and colleagues analyzed long-term outcomes of patients with localized PCa and reported no significant difference in CSS and OS between patients with ePLND and sPLND^19^. When compared to patients who did not receive PLND, no consensus has been reached to support the use of ePLND in terms of survival outcomes. Some researchers demonstrated that a greater number of lymph nodes removed was associated with improved survival^20,21^. In contrast, Liss and colleagues found that patients with ePLND had no better oncological outcomes than patients without PLND^5^. Furthermore, an increased rate of complications was noted in patients with more extensive PLND^5,22^.

Several potential reasons might explain the negative findings in our study. The therapeutic benefit of PLND lay in providing improved survival outcomes by eliminating micro-metastases. Unfortunately, even with ePLND, it was still unlikely to detect and dissect all positive lymph nodes and the remaining unremoved positive nodes would eventually lead to recurrence and progression. It has been reported that only 63% lymph nodes located in the region of ePLND and about 13% positive nodes would have been missed with ePLND^23,24^. Meanwhile, as mentioned above, patients who underwent PLND had more aggressive clinicopathologic characteristics than patients without PLND. It was reasonable to hypothesize that patients with PLND might harbor higher risk of extracapsular extension, seminal vesicle invasion or positive surgical margin which had a negative impact on prognosis and diluted the effect of PLND. In addition, the information on adjuvant therapies was not available for the included patients due to the limitation of SEER database. Thus, it was possible that bias in adjuvant treatments might have an influence on survival and result in negative outcomes of PLND.

Increasing studies have indicated that improved radiological staging techniques such as ^68^Ga-PSMA-PET, were accurate in detecting LNI^25,26^. Moreover, neoadjuvant chemohormonal therapy followed by RP showed superiority in oncological outcomes and cost-effectiveness to RP plus ePLND. Therefore, unclear therapeutic effect, improved radiological techniques and various multimodal treatment approaches forced us to reconsider the necessity of PLND in patients with localized PCa.

There were several strengths in the present study. First, this was a population-based study reflecting the current situation in clinical practice. Second, baseline characteristics were well balanced using PSM. Third, extent of PLND was investigated and subgroup analyses were conducted, which allowed comprehensive understanding of the therapeutic role of PLND. However, this study was not devoid of limitations. As a retrospective study, selection bias was inevitable even with PSM. Besides, the SEER database could not provide all the pathologic data, related complications and subsequent treatments, which could not be adjusted in the analyses. In addition, the extent of PLND in our study was defined by NRN and could not represent the real scale of PLND. Finally, the follow-up period was relatively short and long-term outcome were needed to verify our results.

In conclusion, neither PLND nor its extent was associated with survival in North American patients with localized PCa who had a risk of LNI >5% according to 2012-Briganti nomogram. The cut-off point of 5% probability of LNI might be too low to show benefits in survival in patients underwent PLND. Finding optimal candidates who could benefit from PLND is still challenging, new criteria with high LNI predictive accuracy need to be further explored in the future.

## Materials and Methods

### Study population

Patients with histologically confirmed adenocarcinoma of the prostate (International Classification of Disease for Oncology [61.9]; histological code: 8140) who received RP as primary treatment between 2010 and 2015 in Surveillance, Epidemiology, and End Results (SEER) database were reviewed. Only patients with complete clinicopathological data including clinical T (cT) stage, pathological T (pT) stage, baseline prostate specific antigen (PSA) value, Gleason score (GS) at biopsy, GS at RP, number of positive or negative cores and lymph node status. Exclusion criteria included cT4, PSA >50 ng/ml, probability of LNI ≤5%, metastatic diseases and neoadjuvant therapies.

### Statistical analyses

Probability of LNI was calculated according to 2012-Briganti nomogram recommended by EAU guidelines^7^. Medians and ranges were used to describe continuous variables, proportions were reported for categorical variables. The chi-square tests were used to compare the baseline characteristics. Propensity score matching (PSM) was used to minimize selection bias with a caliper distance of 0.001 based on all baseline characteristics. Age, race, D’Amico risk stratification, cT stage, pT stage, PSA value, GS at biopsy and RP were used in PSM. Study endpoints consisted of cancer-specific survival (CSS) and overall survival (OS), which were defined according to SEER database. Kaplan-Meier method and log-rank tests were used to illustrate and compare the CSS and OS in patients with and without PLND. In addition, patients were further divided according to extent of PLND. Number of removed nodes (NRN) ≥75th percentile was regarded as more extensive PLND. Subgroup analyses were performed to evaluate the prognostic value of PLND in patients with different baseline factors in post-PSM cohort and presented by forest plots. Univariate and multivariate Cox regression analyses were conducted to assess the predictors of CSS and OS in post-PSM cohort.

SPSS version 25.0 (SPSS Inc., Chicago, IL, USA) was used for statistical analyses. All tests were 2-sided with p values < 0.05 considered statistically significant.

### Ethical approval

This retrospective study was approval by Ethics Committee of the West China Hospital, Sichuan University.

### Informed consent

The institutional ethics committee waived informed consent given the retrospective, de-identified nature of the study using SEER data.

## Data Availability

All data generated or analysed during this study are available from SEER databases.
